# Action Experience Changes Attention to Kinematic Cues

**DOI:** 10.3389/fpsyg.2016.00019

**Published:** 2016-02-15

**Authors:** Courtney A. Filippi, Amanda L. Woodward

**Affiliations:** Department of Psychology, University of Chicago, ChicagoIL, USA

**Keywords:** action anticipation, infancy, motor resonance, motor experience, social cognition

## Abstract

The current study used remote corneal reflection eye-tracking to examine the relationship between motor experience and action anticipation in 13-months-old infants. To measure online anticipation of actions infants watched videos where the actor’s hand provided kinematic information (in its orientation) about the type of object that the actor was going to reach for. The actor’s hand orientation either matched the orientation of a rod (congruent cue) or did not match the orientation of the rod (incongruent cue). To examine relations between motor experience and action anticipation, we used a 2 (reach first vs. observe first) × 2 (congruent kinematic cue vs. incongruent kinematic cue) between-subjects design. We show that 13-months-old infants in the observe first condition spontaneously generate rapid online visual predictions to congruent hand orientation cues and do not visually anticipate when presented incongruent cues. We further demonstrate that the speed that these infants generate predictions to congruent motor cues is correlated with their own ability to pre-shape their hands. Finally, we demonstrate that following reaching experience, infants generate rapid predictions to both congruent and incongruent hand shape cues—suggesting that short-term experience changes attention to kinematics.

## Introduction

The ability to anticipate others’ actions allows us to interact with our social partners effectively. By proactively shifting gaze toward the end point of an action before that action is complete, we can efficiently coordinate our actions with others. Research suggests that the ability to anticipate the actions of social partners begins to emerge in infancy and may be coupled with one’s ability to produce these actions oneself (Gredebäck and Kochukhova, 2010; Kochukhova and Gredebäck, 2010; Kanakogi and Itakura, 2011; Cannon et al., 2012; Falck-Ytter, 2012; Ambrosini et al., 2013). Despite considerable interest in the link between action experience and action anticipation, it remains unclear how the motor system translates different experiences into predictions about others’ actions—particularly early in life when the motor system is changing rapidly. The current study examines the effects of reaching experience on action anticipation.

Action experience happens on multiple time scales: across minutes, hours, months, and even years. To date, studies have investigated the relation between action experience and action anticipation by examining experience across two timescales: long term, developmental timescale (across months) and immediate experience (across minutes) timescale. The developmental timescale compares infants who have acquired one skill level to those who have acquired another (e.g., comparing walkers to crawlers). In comparison, research investigating the role of immediate experience examines whether providing action experience immediately before test (typically referred to as motor priming) changes action anticipation. Research has shown that adults and infants are influenced by experience across both timescales.

Across the developmental time scale, research has shown that long-term experience (or expertise) performing an action changes how rapidly both infants and adults predict action events: those with more experience tend to anticipate the timing of others’ actions more accurately (e.g., Aglioti et al., 2008; Stapel et al., 2016) and generate faster visual anticipations to the action endpoint (Gredebäck and Kochukhova, 2010; Kochukhova and Gredebäck, 2010; Daum and Gredebäck, 2011; Kanakogi and Itakura, 2011; Cannon et al., 2012; Ambrosini et al., 2013). For instance, infants with more experience grasping objects generate faster visual anticipations to grasping actions but not back of hand actions or mechanical claw actions—suggesting a correspondence between action prediction and motor development (Kanakogi and Itakura, 2011). The experience of reaching for objects continues to develop throughout infancy as infants acquire fine motor skills and reach more efficiently for objects. Ambrosini et al. (2013) investigated whether these additional developments in fine motor control (specifically in the ability to use a precision grip to grasp small objects) are correlated with action anticipation. Infants observed a person reach for one of two balls using either a whole-hand grip or a precision grip. Following action observation, they tested infants’ own fine motor skills. They found that infants who used fewer fingers to grab small objects generated faster visual predictions to others’ precision grip actions—suggesting that infants’ fine motor ability is linked action anticipation. Thus, across a developmental timescale more experience performing an action is correlated with faster action anticipation.

While these findings show that long-term experience is related to infants’ visual anticipation of actions, as yet, it is not known whether (or how) immediate experience affects action anticipation. Studies that have looked at global levels of attention indicate that there are effects of immediate experience on action perception. To illustrate, Sommerville et al. (2005) gave 3-months-old infants experience coordinating their gaze and manual contact for the first time either before or after testing infants’ sensitivity to others’ goals. They found that only those infants who received this action experience first, show global attention differences in response to the goal structure of others’ actions. This finding and others like it (Hauf et al., 2007; Sommerville et al., 2008; Gerson and Woodward, 2012, 2013) suggest action priming can change some aspects of infants’ attention to others’ actions. While global measures of visual attention provide information at a gross-level of description, they do not provide information about changes in attention as events unfold.

To date, it remains unclear whether (in addition to global attention differences) action priming also affects fine-grained aspects of online visual attention. Two studies have examined the effects of action priming on one measure of fine-grained visual attention (i.e., infants’ online action anticipation) and the findings are mixed. Gredebäck and Kochukhova (2010) tested action anticipation to puzzle actions before or after infants put together puzzles themselves. They found no differences across testing orders—suggesting that some types of experience may not influence infants’ anticipation of others’ actions. In contrast, Cannon et al. (2012) found some evidence that prior experience influenced infants’ action anticipation. Specifically, (although they didn’t find group level improvement following action priming) they found that the amount of action infants engaged in prior to the action observation task influenced how rapidly they anticipated others’ actions (but only among those infants who acted first)—that is, infants who put more toys into the bucket generated faster predictions to the bucket during the subsequent action observation phase.

The current study was designed to address why we find these different effects of experience on action anticipation. We examined the effect of experience across these two timescales by systematically varying the infants’ own experience prior to action observation. Infants were either assigned to engage in a reaching task before (i.e., reach first condition) or after (i.e., observe first condition) the action observation phase. We reasoned that the reach first condition would provide information about the immediate effects of action on visual anticipation. In contrast, the observe first condition would provide information about differences in spontaneous action anticipation as a function of developmental variability in motor skill.

As a test case, we also assessed one aspect of infants’ motor skill: infants’ own hand pre-shaping ability. By 13-months infants’ own reaching behavior is anticipatory (von Hofsten and Ronnqvist, 1988; Claxton et al., 2003). For example, infants pre-shape their hands in anticipation of the size, shape, and orientation of objects before making contact with those objects (Lockman et al., 1984; von Hofsten and Fazel-Zandy, 1984; von Hofsten and Ronnqvist, 1988; Morrongiello and Rocca, 1989). Hand pre-shaping is both a motor behavior that infants engage in and a behavior that (during action observation) could provide information about the type of object a person is reaching for. As such, we expected that 13-months-old would be adept at using this kinematic cue to generate visual predictions about reaching events.

To determine whether 13-months-old infants recruit kinematic details of others’ action to generate action predictions we designed an action observation task where infants observe one of two types of reaching events: either the orientation of an actor’s hand matches the orientation of the object that the hand makes contact with (i.e., congruent reach) or the orientation of the hand fails to match the target object (i.e., incongruent reach). Previous research has compared action anticipation when kinematic cues are present (e.g., hand pre-shapes into a precision grip) vs. absent (e.g., fist reaches toward object; see Ambrosini et al., 2013). We reasoned that our task could be more challenging because in the incongruent reaching event, the hand pre-shaping information matches one object on the screen yet the actor always reaches for the object that is incongruent with hand pre-shaping. We hypothesized that 13-months-old infants would spontaneously generate faster visual predictions when the target could be predicted by (congruent cue) the hand pre-shaping than when the target could not be predicted by (incongruent cue) hand pre-shaping.

The first aim of the current study is to evaluate whether infants’ own hand pre-shaping is correlated with their recruitment of kinematic cues independent of their immediate experience. To do so, we also assess infants’ own hand pre-shaping ability by giving these infants the opportunity to reach for a toy after the action anticipation task (Observe first condition). We reasoned that by recruiting variability across a developmental timescale we could examine whether there is a relationship between spontaneous attention to others’ actions and infants’ own motor skill. In line with previous research, we hypothesized that infants who spontaneously pre-shaped their hand more (in the observe first condition) would generate faster visual predictions when the kinematic cue was congruent but that this relationship would not be found in the incongruent kinematic cue condition. This would provide converging evidence that infants draw on their experience pre-shaping their hands when they recruit kinematic cues to anticipate others’ actions—particularly when kinematic cues are present and reliable.

Our second aim was to assess whether immediate action experience changes attention to kinematic cues. To do so, we gave infants the opportunity to reach for a toy before (Reach first condition) we assessed their action anticipation. We hypothesized that if immediate action experience facilitates attention to kinematics, then infants in the reach first condition would generate faster congruent predictions than the infants in the observe first condition because their motor system is already primed to attend to kinematics. Alternatively, if immediate action experience facilitates attention to goal, infants in the reach first condition may generate equally fast predictions on congruent and incongruent trials.

## Materials and Methods

### Ethics Statement

The Institutional Review Board at the University of Chicago approved the protocol for this study and written consent was provided by infants’ parents/legal guardians prior to participation.

### Participants

Participants were 70 full-term 13-months-old infants (36 females, *M* = 13 months, 2 days, *SD* = 9 days, range = 12; 12–13; 21) recruited from a large metropolitan city. Half of the infants (*n* = 36) were randomly assigned to do the eye-tracking task before the reaching task (Observe first condition) and half of the infants (*n* = 34) were randomly assigned to perform the reaching task before watching the eye-tracking videos (Reach first condition). 44% of infants were European American, 25% were African American, 3% were Asian, 10% were Hispanic, and 17% were mixed ethnicity. An additional 46 infants were tested but excluded from analyses due to fussiness during eye-tracking (*n* = 9), equipment failure (*n* = 7), failure to calibrate or percent data collected less than 50% (*n* = 11), fewer than three trials of predictive looks (*n* = 18^1^) and refusal to participate in the behavioral task (*n* = 1).

### Apparatus and Stimuli

Data were collected via corneal reflection using a Tobii T60 XL eye-tracker (accuracy 0.5°, sampling rate 60 Hz) with a 24^′′^ monitor, from a viewing distance of ∼60 cm. Infants sat on their parents lap and parents were asked not to direct infant’s attention during testing.

**FIGURE 1:**
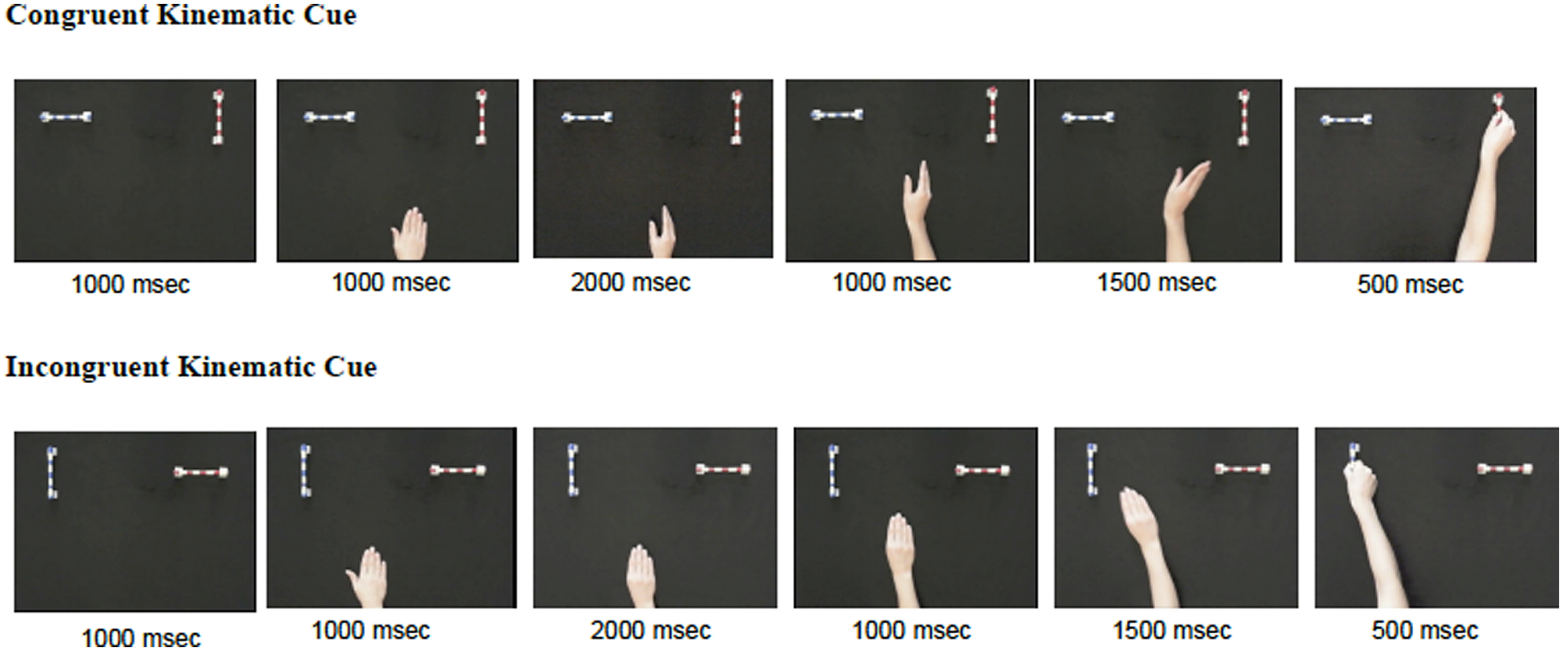
**Depiction of congruent (top) and incongruent (bottom) video events.** These are two examples taken from a set of four possible congruent reaches and four possible incongruent reaches. Listed below each screenshot is the event duration. From left to right: Only objects present, hand enters scene flat on the table, hand forms shape and pauses, hand moves forward equidistant between the objects, hand deflects toward one of the objects, hand grasps object and pauses.

Infants watched a short video of a hand reaching for one of two rods. See **Figure 1** for screenshots of each phase of the video as outlined below. These rods always remained in the same location (i.e., the blue rod was always on the left and the red rod was always on the right). The videos were timed such that infants were given 1000 ms to notice the rods before the hand entered the scene. After this time, the hand entered the scene flat on the table (event duration: 1000 ms). The hand then formed a shape and paused in that shape for 2000 ms. While retaining this shape, the hand then moved forward equidistant between both rods (event duration: 1000 ms). The hand continued in a smooth motion deflecting toward one of the two rods until it contacted that target rod (event duration: 1500 ms). Once the hand grasped the target rod it paused in this position for 500 ms.

### Procedure

#### Action Observation Task

Eye-tracking began with a nine-point calibration period, followed by two blocks of videos. Each block consisted of six identical trials in which a hand reached (once per trial) for an object using a hand shape that was congruent with the target object (i.e., the object that the hand ultimately grasps) or incongruent with the target object. Each infant received one block of congruent trials and one block of incongruent trials, with the order of trial blocks counterbalanced across infants. Pilot data indicated order effects; as such we do not report data from the second block here.

In the first block of trials, infants either watched one of four possible congruent reaches (congruent cue) or one of four possible incongruent reaches (incongruent cue). Congruent reaches always correctly anticipated the orientation of the rod before the midpoint of the reach (see **Figure 1**). In contrast, incongruent reaches failed to match the orientation of the rod up until the hand was about to make contact with it, and the initial posture of the hand was appropriate for the non-target object (see **Figure 1**). Across infants, the order of the blocks, the side reached to, the hand shape, and the orientation of the objects were counterbalanced.

#### Reaching Task

Either before (Reach first condition) or after (Observe first condition) the eye-tracking task, infants were encouraged to reach for a rod presented by an experimenter. The rod was presented ∼19 cm from the infant. The experimenter presented the rod in one of two orientations (horizontal or vertical). The order of presentation was constant for all infants. The experimenter first presented the rod in a horizontal orientation for five trials, then oriented the rod vertically for five trials, then alternated between horizontal and vertical orientation every trial thereafter. The experimenter presented the rod to the infant until they became fussy or lost interest. On average infants reached 19.48 times (*SD* = 7.932, range = 3–49)^2^.

### Eye-Tracking Data Reduction

Data were exported using the Tobii Fixation filter with the strict average eye selection criteria. Fixations were classified using 35 pixels/window velocity and distance threshold.

#### Areas of Interest (AOIs)

The current study only examines the timing of fixations that were directed toward the hand and objects areas of interest (AOIs; these AOIs are made visible in Supplementary Figure S1). The target object AOI was defined as the object that the hand ultimately reaches for, whereas the distractor object is the untouched object. These AOIs are ∼5° of visual angle off center. These AOIs were found in pilot testing to capture most visual fixations toward the object. Participants were unaware of these regions of interest as they were only present during the data reduction process.

#### Coding Criteria

In order to determine whether a look to the target AOI (or distractor AOI) was indeed generated based on attention to hand shape, we established the following criteria for all visual fixations to be included in this dataset: (1) infants had to first fixate within the hand AOI, (2) this fixation to the hand had to occur after the hand began to form its shape, (3) infants next fixation had to be toward one of the two objects. We recorded the time of first looks to both the target and distractor objects.

#### Latency to Predict the Target Object

Latency scores were determined by subtracting the time that the hand was outside of the target object AOI (see Supplementary Figure S2) from the time of the first visual fixation to the target object. Average latency scores were used to assess how rapidly infants visually anticipated the actions of others. Average latency scores that exceeded 2.5 SD from the group mean (*n* = 1) were removed from subsequent analyses.

Looks to the target object that occur after the hand enters the target AOI are considered reactive. Compared to other work on infant action anticipation, this is a rather conservative measure of which looks are anticipatory. Given this scoring system, negative values represent prospective looks to the target object, 0 is the time that the hand enters the object AOI, and positive values represent reactive looks to the target object.

#### Global Measures of Attention

Attention was also measured by assessing the duration of time that infants looked to the target object AOI, the distractor object AOI, and the hand AOI. We evaluated total attention to the event with a whole screen AOI and we also analyzed attention to each AOI separately. All summary statistics are computed as an average across all trials.

#### Distractor Predictions

Since action observation events provided hand shape cues that always matched one of the two objects, it is possible that infants that observe an incongruent cue would be more likely to generate predictions to the distractor object. To test whether there were differences in infants’ propensity to generate first predictions to the distractor, we analyzed the proportion of trials that each infant generated a predictive look to the distractor first. We averaged distractor predictions across all trials to create an average proportion of distractor predictions score.

### Behavioral Data Reduction

We also coded infant reaching behavior during the motor behavior task to determine whether hand pre-shaping is related to action anticipation.

#### Hand Pre-shaping during Reaching

**FIGURE 2:**
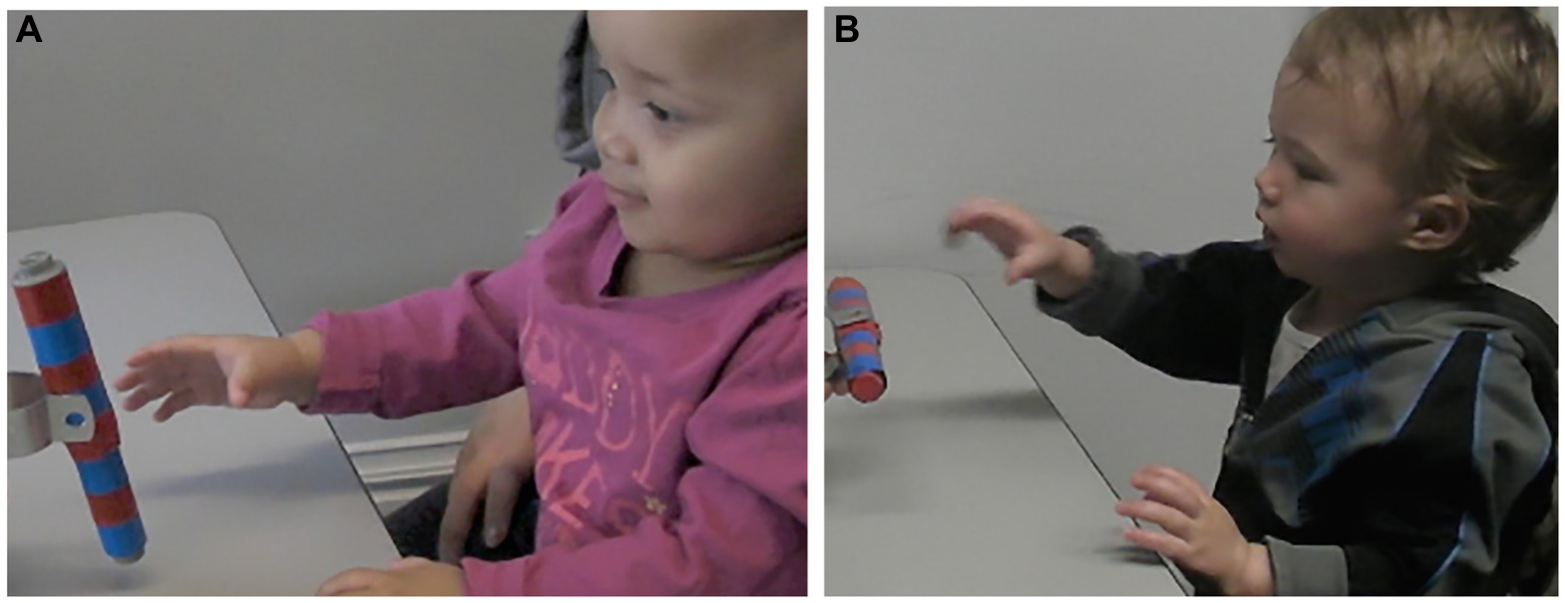
**Still image of infant hand pre-shaping behavior on horizontal rod orientation trials **(A)** and vertical rod orientation trials **(B)** of the motor behavior task**.

To examine the kinematics of infants’ own movement, on each reaching trial we coded whether infants pre-shaped their hand to match the orientation of the target object prior to contact with the object (see **Figure 2**). Coding was performed oﬄine using Interact, a digital coding program (Mangold, 2010). The initiation of the reach was identified as the first frame when the infant moved toward the rod. The end of the reach was identified as the time when the hand first touched the rod. Since infants could interact with the object any way they wished on each trial, we eliminated data from any trial where the infants’ goal was not to grasp the object and trials where the hand shape was identified as ambiguous such that the coder could not identify whether it was a match or not. We found that on average infants pointed to the object instead of reaching on 0.314 (*SD* = 0.692, range = 0–3) trials and infants acted in a way that we couldn’t identify as goal-directed on average 1.59 (*SD* = 1.63, range = 0–7) trials. After eliminating trials where the infant did not grasp the toy, we computed an average score indicative of the proportion of trials that the infants pre-shaped their hand to match the orientation of the rod as they reached. A second independent coder coded 25% of infants and the two coders were in agreement on 93% of trials.

## Results

In the design of the experiment, the testing orders provide information about two different timescales: developmental time scale and immediate experience time scale. The observe first condition, provides information about the relationship between spontaneous action anticipation and the kinematics of infants’ own actions. In contrast, the reach first condition can tell us how immediate experience changes action anticipation. Below we present analyses to examine infants’ attention to action kinematics and the relationship between infants’ own actions and their anticipation of actions they observe. We begin with the observe first condition. Then, we present data from the reach first condition. Finally, we investigate similarities and differences between the two conditions to assess the effect of immediate experience on visual attention and infants’ own reaching behavior.

Preliminary analyses indicated no reliable effects of gender, age (as a covariate), number of trials infants reached during action task (as a covariate), whether the hand reached to the right or left, handshape (horizontal vs. vertical grip) or rod orientation (horizontal vs. vertical) or the number of visual predictions generated (all *p*s > 0.111) on gaze latency. However, there was a main effect of the orientation of the target object [*F*(1,62) = 3.984, *p* < 0.050] on gaze latency—indicating that infants generated faster visual predictions to the vertically orientated target. This is unsurprising given that the vertical object AOI extends down closer to the hand than the horizontal object. Importantly, there were no interactions between target object orientation and condition (reach first vs. observe first) or cue (congruent vs. incongruent). Therefore, these factors were not included in subsequent analyses.

### Observe First Condition

**FIGURE 3:**
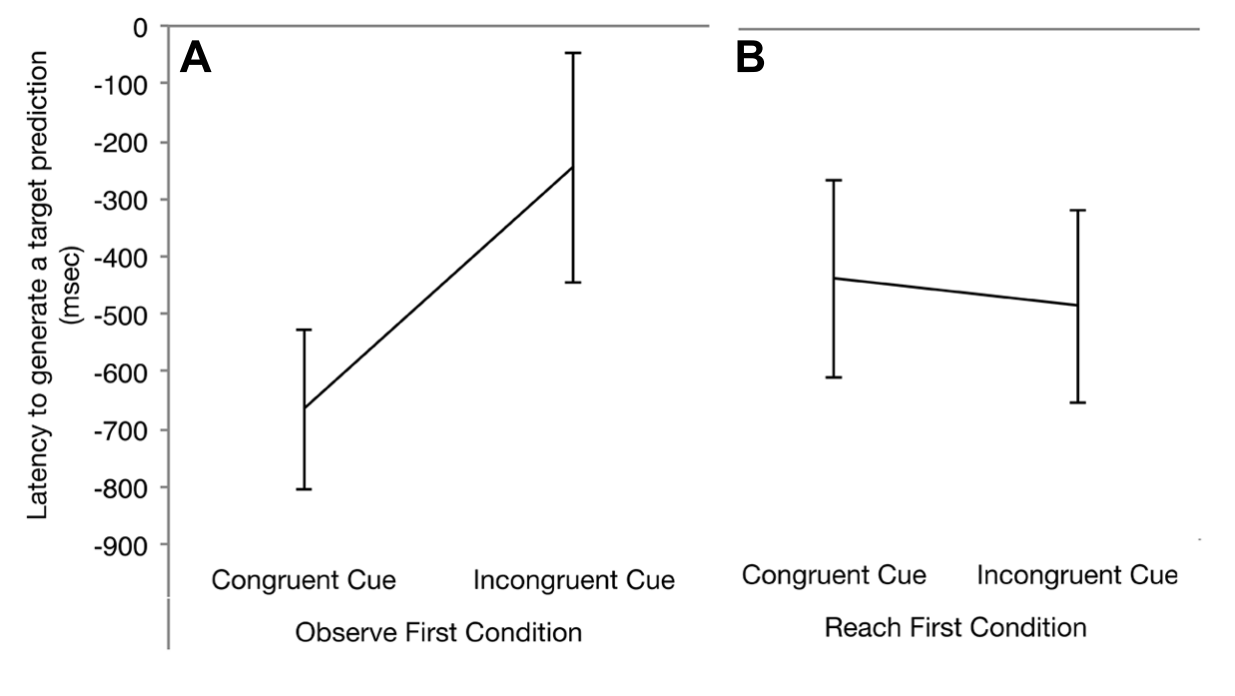
**Average mean latency scores for infants in the observe first condition **(A)** and reach first condition **(B)****.

**Figure 3A** summarizes gaze latency scores across cue type (congruent vs. incongruent) for the Observe First condition. To begin, we asked whether infants reliably anticipated the hand’s arrival to the target before the hand entered the target AOI. To determine whether infants reliably anticipated the hand’s arrival, we compared average latency scores to 0—the time when the hand enters the target AOI. One sample *t*-test indicated that infants who observed congruent kinematic cues generated rapid saccades to the target and these looks to the target arrived before the hand entered the target object AOI [*t*(17) = –4.728, *p* < 0.001]. In comparison, infants that viewed incongruent reaches did not look to the target before the hand entered the target AOI [*t*(17) = –1.244, *p* < 0.230]. An independent samples *t*-test was conducted on latency scores with trial type (congruent vs. incongruent) as the between subjects factor. Based on prior research, we also expected that infants would make faster predictions when hand pre-shaping matched the target object. Consistent with previous research, we found that gaze latency scores for congruent cues were faster compared to incongruent cues [*F*(1,34) = 3.214, *p* < 0.041, one-tailed]. Together, these findings suggest that infants spontaneously recruit kinematic cues to generate visual predictions.

In the action observation events the hand shape always anticipates one of the two objects. As such, it’s possible that infants that watched incongruent reaches were more likely to generate predictions to the distractor object than infants that watched congruent reaches. If so, this could suggest that infants have difficulty ignoring kinematic cues. To test whether this was the case, *Z*-test of two population proportions was conducted on the proportion of distractor predictions with cue type as the between subjects factor. We found that infants did not differ in the proportion of distractor predictions generated (*Z* =-0.209, *p* > 0.834, congruent cue *M* = 0.152, *SD* = 0.243; incongruent cue *M* = 0.150, *SD* = 0.189). In combination with the gaze latency findings, this suggests that incongruent kinematic cues did not lead infants to produce wrong guesses about the target object. Nevertheless, saccades to the target were slower on incongruent compared to congruent trials. We suspect that this may be due to the availability of other cues (e.g., direction of motion) and because the trial always ended with the hand grasping one of the objects.

**FIGURE 4:**
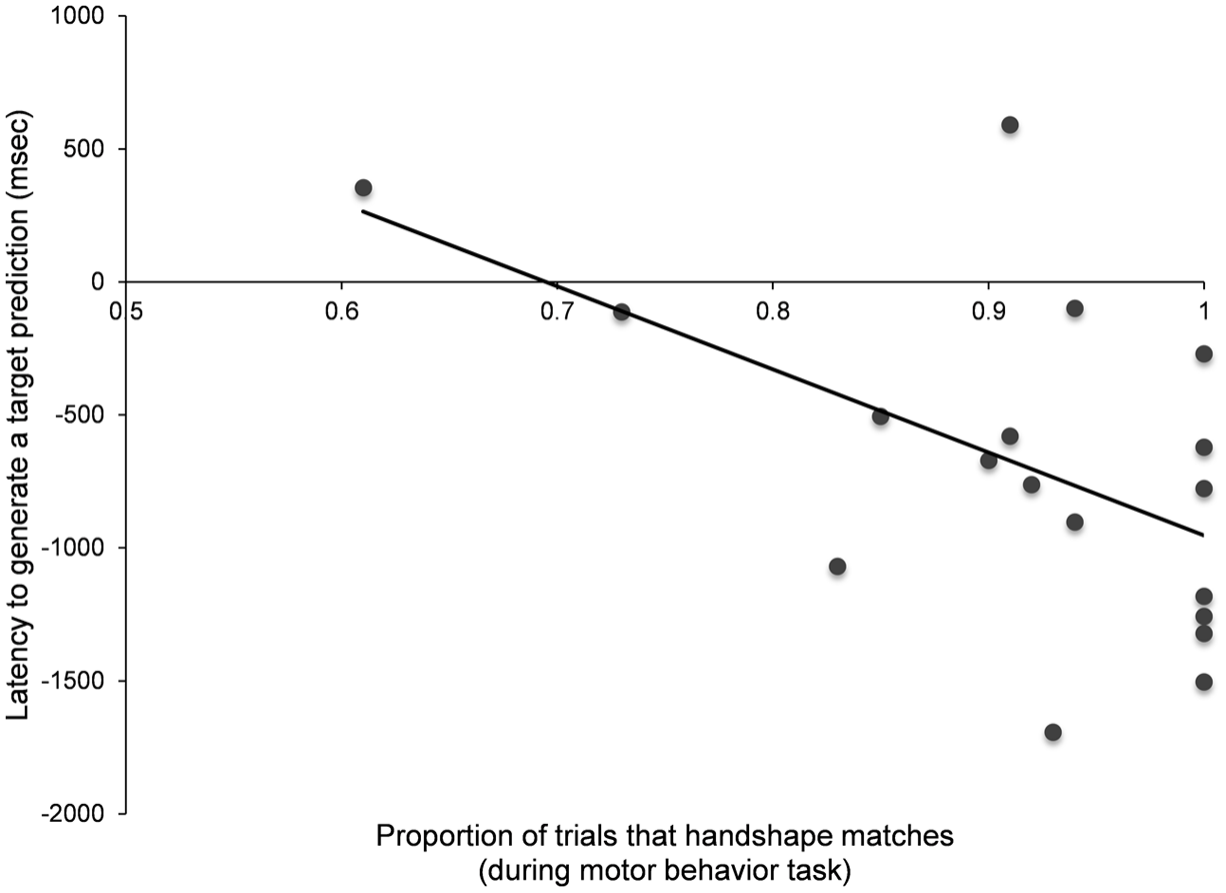
**Observe first- congruent cue condition.** Mean gaze latency as a function of hand pre-shaping behavior.

We next evaluated whether hand pre-shaping behavior correlated with how rapidly infants generated visual predictions. In line with previous research (Ambrosini et al., 2013), we found that the proportion of trials where infants’ hand shape matched the orientation of the rod during the reaching task was correlated with how rapidly infants generated visual predictions (*r* = –0.541, *p* < 0.021) on congruent trials—that is, more hand pre-shaping behavior predicted faster visual predictions on congruent trials (see **Figure 4**). To examine whether this effect was driven by some infants being more motivated to reach for toys, we tested whether this relationship held when controlling for the number of times infants reached in the motor behavior task. We found that even after controlling for the number of trials infants reached, this effect remained significant (*r* = –0.622, *p* < 0.008). Critically, we found that this relationship was selective. Infants that viewed incongruent cues did not show this correlation (*r* = 0.344, *p* < 0.177). These findings suggest that motor experience is selectively linked to generating predictions when kinematic cues are present and reliable—not to actions where the target is incongruent with kinematic cues.

These findings are concordant with a body of research (Ambrosini et al., 2011, 2013; Kanakogi and Itakura, 2011) demonstrating that motor skill is linked to action anticipation. In the next section, we test whether we see similar patterns of behavior following immediate action experience.

### Reach First Condition

Next we examined whether immediate reaching experience changes recruitment of kinematic cues to generate visual predictions. **Figure 3B** summarizes gaze latency scores across cue type (congruent vs. incongruent) for the Reach First condition. To begin, we assessed whether infants reliably predicted the target. One sample *t*-test indicated that infants who observed congruent cues [*t*(18) = –2.527, *p* < 0.021] and incongruent cues [*t*(17) = –2.877, *p* < 0.010] generated predictive saccades that entered the target AOI before the hand. To determine whether gaze latency differed across cue type, we conducted an independent samples *t*-test on gaze latency with cue type (congruent vs. incongruent) as a between subjects factor. We found no significant effect of cue type [*t*(32) = 0.377, *p* < 0.708]. These findings suggest that infants who received reaching experience immediately before action observation generated rapid visual anticipations regardless of cue type.

Mean gaze latency as a function of hand pre-shaping behavior.

Follow-up analyses indicated that (just like infants in the observe first condition) infants in the reach first condition did not show a difference in the proportion of distractor predictions across congruent (*M* = 0.202, *SD* = 0.281) and incongruent cue (*M* = 0.247, *SD* = 0.292) type (*Z* = –0.788, *p* > 0.430). Again suggesting that infants were able to generate predictions to the target object regardless of cue. We next asked whether the manner in which infants reached and grasped the toy was related to gaze latency. To do so, we examined the relationship between hand pre-shaping and gaze latency. We found no correlation between gaze latency and hand pre-shaping (*p*s > 0.198)—suggesting that planning one’s own actions was not related to predicting others’ actions.

### Comparing Observe First and Reach First Conditions

To determine whether there were any group differences in motor behavior or visual attention that could account for differences in performance between our reach first and observe first conditions, we ran follow up analyses to compare the groups.

#### Motor Behavior Task Performance

We might find differences in infants’ performance on the action observation task because infants in the observe first condition may have more advanced motor skills than those infants in the reach first condition or because reaching early or late in the testing session may result in differences in behavioral performance. To assess whether this was the case, we used an independent samples *t*-test to compare the proportion of trials where infants pre-shaped their hands in the motor behavior task across conditions (observe first vs. reach first). Results indicated no significant difference in the proportion of trials with hand pre-shaping across the reach first (*M* = 0.89) and observe first conditions [*M* = 0.91; *t*(67) = –0.706, *p* < 0.483].

#### Comparing Visual Attention to the Action Observation Events

We could have found differential recruitment of kinematic cues across conditions if infants in the reach first condition were not attending to the action observation videos as much as infants in the observe first condition or if reaching prior to action observation resulted in fatigue during the action observation session. To examine these possibilities, we next conducted a one-way ANOVA on total attention with condition (reach first vs. observe first) and cue type (congruent vs. incongruent) as factors. We found no difference in how long infants attended to the action events across condition (observe first vs. reach first), cue type (congruent vs. incongruent) and no significant interaction (*p*s > 0.372). To further assess whether allocation of attention differed across conditions, we examined whether infants attended to all parts of the action events equally. The two groups did not vary in allocation of attention to the hand (*p*s > 0.255), target object (*p*s > 0.234), or distractor object (*p*s > 0.297). Thus, infants attended equally to all aspects of the action events.

#### Gaze Latency

Analyses within condition (reach first vs. observe first) suggest that there are differences in gaze latency. As a way to evaluate how the latencies across conditions are related to one another we compared latency scores across both conditions. To assess whether there were significant differences in gaze latency across the two conditions we conducted an univariate ANOVA on gaze latency with condition (reach first vs. observe first) and cue type (congruent vs. incongruent) as between subjects factors. We found no significant differences in gaze latency across condition (*p* > 0.973) or cue type (*p* > 0.346) and no interaction (*p* > 0.138). This suggests that while we found differences in the relative speed at which infants generated predictions in the observe first condition, we do not find that these differences are significantly different from those infants in the reach first condition.

Overall, these findings suggest that infants can recruit kinematic cues to generate action predictions. Furthermore, infants spontaneously recruit their own motor skill to generate action predictions. We also found that the experience of reaching for objects changes action prediction: when provided experience reaching for objects prior to action observation, we find that infants generate equally fast predictions to congruent reaches and incongruent reaches. This effect is not driven by low-level attention to the observed stimulus and cannot be accounted for by the number of trials that infants reached for the toy.

## Discussion

The current study examined the relationship between action experience and action anticipation. Infants were randomly assigned to either observe actions before (Observe first condition) or after (Reach first condition) a motor task. To assess action anticipation we used a novel paradigm that varied the action observation event in terms of whether the hand pre-shaping did (congruent cue) or did not (incongruent cue) predict the orientation of the target object. Consistent with prior research, we found that infants who observed the action events first (Observe first condition) recruited kinematic cues to generate predictions. Additionally, we found that infants’ own hand pre-shaping behavior predicted how rapidly they generated predictions when the kinematic cue was congruent with the target (see Ambrosini et al., 2013 for similar findings). In comparison, infants who engaged in a motor behavior task before observing action events (Reach first condition) generated rapid visual predictions to both congruent and incongruent kinematic cues. Together, these findings suggest that action experience across different time scales may influence action anticipation differently.

### Action Anticipation: The Developmental Timescale Perspective

The observe first condition findings provide converging evidence for the claim that infants recruit kinematic cues when they are available (Ambrosini et al., 2013) and that there may be a correspondence between infants’ motor abilities and anticipation of others’ actions (Kochukhova and Gredebäck, 2010; Kanakogi and Itakura, 2011; Cannon et al., 2012; Ambrosini et al., 2013). Our design also expands upon this body of work by testing action anticipation when the motor cue is incongruent with the target object. Previous research has shown that infants generate faster covert shifts in the direction of a hand’s opening compared to when an object appears to be incongruent with a hand’s opening (Daum and Gredebäck, 2011). In contrast, the current study shows that infants use hand orientation information to generate online visual anticipations when an actor is choosing between two objects. Our findings harmonize with previous research—both studies show that infants are faster to generate predictions on congruent trials compared to incongruent trials. Further, we show that on incongruent cue trials, infants generated looks to the target that (on average) arrived at approximately the same time that the hand made contact with the rod. This suggests that the tendency to recruit kinematic information may be difficult to override.

By including incongruent reaching events, we were able to assess the tendency to recruit kinematic information when this information is incompatible with the target object. Our incongruent events were perceptually identical to the congruent events up until the moment that the hand made contact with the toy. Thus, differences in action anticipation were due to attention to the relationship between the kinematics of the observed action and the target objects orientation. This paradigm allowed us to assess infants’ tendency to use kinematic information on incongruent trial events by examining infants’ propensity for generate predictions to the distractor object. We show that when infants observed events where hand pre-shaping is incongruent with the target, infants, nevertheless, generate predictions to the target. We suspect that this may be because the incongruent reaches that infants observe always result in the hand grasping one of the two toys. After a demonstration of this actor’s preference, infants may override their processing of the kinematic cue to generate a target prediction. If the reach was never completed, we may not have found such a strong propensity to generate target predictions. Future work is needed to examine this possibility.

### Action Anticipation Following Immediate Experience

Following immediate reaching experience, we found that infants generated rapid predictions to both congruent and incongruent cues. Furthermore, we found that the amount of reaching performed during behavioral testing and the extent to which their own grasping behavior matched the observed action, did not correlate with gaze latency. Our findings also indicated that there were not differences in infants’ global attention to the action observation events. Infants across both conditions (reach first vs. observe first) attended to the action observation videos for similar amounts of time and distributed their attention to the target object, hand, and distractor object AOIs similarly. This suggests that infants’ visual attention to the events was comparable but that the motor behavior task may have primed infants to recruit the information in the action observation videos differently. These findings suggest that immediate experience reaching changes attention to (and use of) kinematic cues. Furthermore, this change may not be due to an overt shift in visual attention to others’ movements. We speculate that action priming may prime attention to the goal structure of others’ actions (rather than drawing attention to the specifics of how an actor moves). We suspect that infants in our study are shifting their attention toward the goal structure of others’ actions following action priming because these infants reliably anticipate the target object on incongruent trials. While our findings primarily speak to the speed of infant’s visual anticipations, it could be that action priming facilitates more rapid interpretation of the action in terms of the actor’s goal or that action priming leads infants to rapidly perceive the actor-goal relation (even in the face of incongruent kinematic information).

The sensorimotor system is organized hierarchically (see Rosenbaum et al., 2004; Grafton and Hamilton, 2007 for review) and as such actions can be described at multiple levels. One interpretation of our reach first condition data could be that immediate action experience may prime a motor representation higher than kinematics. This would be in line with our finding that infants who receive action experience before observation, do not recruit kinematic cues in the same way that infants spontaneously recruit kinematic information. However, it is also possible that infants recruit kinematic information in addition to higher representations of the action goal (or recruit them concurrently) and this leads infants to be able to override their sensitivity to incongruent kinematic cues. Future research is needed to evaluate whether this could be the case.

Research suggests that experts (e.g., expert golfers, soccer players, etc.) tend to pay less attention to the kinematics of their own actions and more attention to their goal. Indeed, when acting, experts’ performance suffers when they attend to the specific movements involved in their action (Beilock et al., 2002; Beilock and Gray, 2012). Similar effects have been found with young infants learning to coordinate their visual and manual actions in sequences. Gerson and Woodward (2013) trained 8-months-old infants on how to pull a cloth to obtain an out of reach toy by either highlighting the means (cloth) or the goal (toy). They found that infants learned more rapidly and sustained this learning throughout training if the training emphasized the goal of the action rather than the means needed to achieve the action (Gerson and Woodward, 2013). Given the close link between action execution and action observation across the lifespan (Kontra et al., 2012), it’s possible that devoting considerable attention to the fine details of movement either during movement or immediately prior to observing someone else could make it more difficult to see the goal structure of an action sequence—particularly early in life. In line with this idea, research has also shown that the experience of coordinating visual and manual actions immediately before observing others act, facilitates attention to others’ goals—not to the manner in which arms move through space (Krogh-Jesperson and Woodward, in preparation; Sommerville et al., 2005; see Woodward et al., 2009 for review). When considered in combination with our reach first condition findings, our work provides converging evidence for this claim. Future research should manipulate action tasks to highlight either the goal or the manner used to achieve the goal and assess effects on action anticipation.

### Limitations

While these results suggest that there are differences across conditions in how reliably infants generate anticipatory predictions to the target, we did not find significant differences in overall gaze latency scores. This suggests that while infants are on average generating predictions to the target before the hand enters the target AOI (in all conditions except when observing incongruent trials in the observe first condition), overall prediction speeds are not significantly different across conditions. This raises a number of questions about the extent to which action priming changes action prediction. Our findings suggests that priming may change the relative speed of action prediction—that is, action priming may help infants reliably generate predictions ahead of hand movement particularly when faced with incongruent kinematic cues. Whereas, infants spontaneous behavior (i.e., observe first condition) suggests that they are likely following the hand’s motion (as they do not generate saccades to the target before the hand enters the target AOI) when faced with incongruent kinematic cues.

One reason that we see no overall differences across conditions may be because there is substantial individual variability in infants’ action prediction speed that is unaccounted for—possibly due to differences in general cognitive abilities (e.g., inhibitory control or speed of processing). Indeed, generating a prediction to the target requires the capacity to inhibit looking at the moving hand. This capacity may be underdeveloped at 13 months and limit the range of latency scores. Future research should examine the factors that could contribute to the large variability found across conditions.

## Conclusion

The current study provides novel insight into the link between action experience and action anticipation. Many studies suggest that action experience (Sommerville and Woodward, 2005; Sommerville et al., 2005; Woodward et al., 2009; Gerson and Woodward, 2014) is at the center of action understanding. This past research tested whether action experience changes infants’ high-level understanding of actions (i.e., that actions are structured by goals; e.g., Flanagan and Johansson, 2003; Sommerville and Woodward, 2010). Our data suggest that comparing these timescales can provide us new information about the mechanism that facilitates rapid anticipatory shifts in attention. We show that infants’ immediate experience changes their recruitment of kinematic cues: following a simple reaching task, infants generated rapid predictions to the target object, regardless of kinematic cue congruency. This is different from how infants spontaneously recruit kinematic information. Without immediate reaching experience, infants appear to use kinematic information to generate predictions and they recruit their own ability to execute this specific motor skill.

In conclusion, these findings provide novel evidence to suggest that different types of action experience (e.g., lifetime vs. immediate) could prime infants to recruit motor cues in different ways. Indeed, our findings suggest that immediate experience may prime attention to action goals rather than kinematics. We suggest that this harmonizes with studies of adult skill expertise and infant action understanding. Together these findings raise new questions about the role that the motor system and action hierarchies may play in the development of action anticipation abilities.

## Author Contributions

CF and AW contributed to the study design and concept. CF collected the data, performed data analysis, and interpreted findings under the supervision of AW. CF drafted the manuscript and AW provided critical revisions.

## Conflict of Interest Statement

The authors declare that the research was conducted in the absence of any commercial or financial relationships that could be construed as a potential conflict of interest.

## Footnotes

1 In our analyses we excluded infants that did not generate three predictive looks because our primary analyses concerned average latency scores. Here, we report the number of participants in each condition that were excluded for this reason: Reach first-Congruent cue: *n* = 3; Reach first-Incongruent cue: *n* = 8; Observe first-Congruent cue: *n* = 3; Observe first-Incongruent cue: *n* = 4. This suggests that infants in the Reach first-Incongruent cue condition were twice as likely to be excluded for not looking to the target.

2 Only 2 infants reached fewer than seven times. All other infants reached at least nine times.
