# Supplementary figures and images for: Action Experience Changes Attention to Kinematic Cues
Source: Front Psychol. 2016 Feb 15;7:19. doi: 10.3389/fpsyg.2016.00019 (PMC4753290; doi:10.3389/fpsyg.2016.00019)

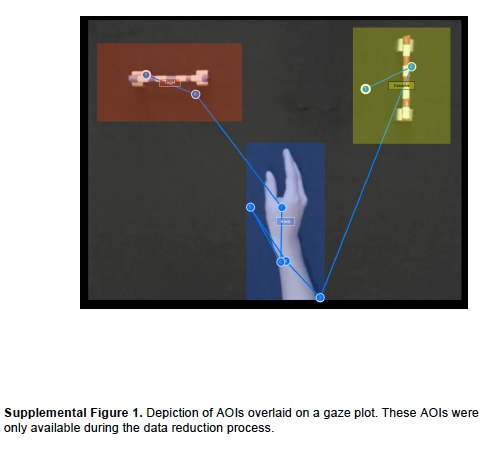

Supplement: Supplementary file 1 [file Image_1.TIFF]

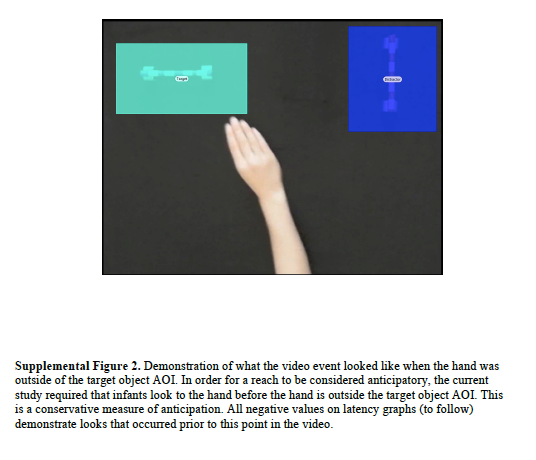

Supplement: Supplementary file 2 [file Image_2.TIFF]
